# Potential disease agents in domestic goats and relevance to bighorn sheep (*Ovis canadensis*) management

**DOI:** 10.1371/journal.pone.0173396

**Published:** 2017-03-10

**Authors:** Mark L. Drew, Glen C. Weiser

**Affiliations:** 1 Wildlife Health Laboratory, Idaho Department of Fish and Game, Eagle, Idaho, United States of America; 2 Caine Veterinary Teaching Center, University of Idaho, Caldwell, Idaho, United States of America; Université de Sherbrooke, CANADA

## Abstract

Domestic goats are raised for meat, milk and hair production, in herds for rangeland weed control, and as pack animals. Domestic sheep, goats and wild bighorn sheep are all susceptible to a multifactorial pneumonia. We sampled 43 herd goats from 7 herds and 48 pack goats from 11 herds for viral and bacterial serology, parasitology, and Pasteurellaceae microbiology. The goats in this study were in generally good health, although most goats did harbor various pathogens and parasites including several bacteria, specifically Pasteurellaceae, which have been associated with pneumonia in free-ranging bighorn sheep. It is not known if domestic goats can transmit the Pasteurellaceae or other pathogens found in this study readily to wild bighorn sheep. However, due the possibility of transmission, domestic goats in areas in or near bighorn sheep habitat should be managed to minimize the risk of spreading disease agents to bighorn sheep.

## Introduction

Domestic goats (*Capra hircus*) are part of the small ruminant livestock population in the United States where they are raised for meat, milk and hair production [1, 2]. Domestic goats are used for or considered for weed control on public and private lands, especially in areas that are difficult to access by motor vehicles. In addition, goats are becoming popular for use in the backcountry as pack animals [1, 2].

Infectious diseases of domestic goats are well studied [3]. One of the most common disease conditions in domestic goats, as well as sheep, is pneumonia [4]. Numerous pathogens have been isolated and considered to be part of the etiology of pneumonia in goats and sheep but the involvement of *Mannheimia haemolytica* is considered important [5]. The cause of pneumonia in domestic sheep and goats is often multifactorial and difficult to pinpoint, even in the presence of pathogens.

Bighorn sheep (*Ovis canadensis*) (BHS) are the only species of native wild sheep in the western United States. Populations of BHS in the United States, once estimated at 2 million, were reduced to about less than 5% of historical levels or about 70,000 animals by 1999 [6]. The decline of BHS populations is probably due to loss of habitat, overhunting, competition for forage with domestic livestock, predation, and disease [7,8]. Respiratory disease appears to be a major limiting factor in BHS population dynamics although the relationship between BHS population density and disease is not clear [9]. The cause of pneumonia in BHS is likely multifactorial [8, 10] with pathogen introduction from domestic livestock that occurred in the past. [11, 12]. However introduction of new pathogens with direct contact with domestic livestock, including goats, is also possible. Recently *Mycoplasma ovipneumonia* has been implicated at a major cause of lamb mortality in BHS [13].

There is some controversy about the relative risk of disease transmission between domestic goats and free-ranging BHS [14, 15]. One co-pasturing study of goats and BHS did not result in respiratory disease [14] but another found that BHS died of respiratory disease after co-pasturing with goats [16]. Domestic pack goats harbor several *Pasteurella* spp. that are considered to be potential pathogens for BHS [17]. The possibility for transmission of disease agents between domestic goats used as pack animals and for weed management and BHS should be part of the management of domestic goats in areas in or near BHS habitat.

The objectives of this study were to 1) evaluate the health status and disease exposure of domestic goats using the same methods as are used for BHS [18], and 2) to use this information to assess risk management for situations in which domestic goats may interact with BHS. Both objectives were attained.

## Materials and methods

### Animals and sampling

Owners of domestic goats used for weed control, pack animals or private pets in southwest Idaho, northeast Oregon, and southwest Washington were contacted for permission to sample animals within individual herds. Pack goats were defined as animals that were used for packing on trails. Herd goats were defined as animals that were pastured or confined on one premise. A total of 91 goats from 18 herds were sampled in 2003 including 48 pack goats from 11 herds and 43 herd goats from 7 herds. The pack goats were exclusively male with 4 intact and 44 castrated animals. Herd goats were predominantly female with 30 females, 12 wethers and 1 intact male. The average age was 7.4 yr for pack goats and 3.1 yr for herd goats.

Goats were physically restrained for physical examination and sample collection. Body condition was assessed by palpation of the topline, ribs and hips and assigned a subjective score of 1 to 5 with 5 being obese. Animal handling and sampling was specifically approved by the University of Idaho IACUC, protocol #2003–25.

### Serology

Blood was collected by jugular venipuncture and placed in sterile glass tubes (Vacutainer, BD Laboratories, Franklin Lakes, NJ). Blood was allowed to clot, centrifuged and the serum decanted. Serum was frozen at -20 C until analysis at the Idaho State Department of Agriculture Animal Health Laboratory, Boise, ID. Standard serological procedures used by the Idaho State Department of Agriculture Animal Health Laboratory or the National Veterinary Services Laboratory, Ames, IA were used to detect antibodies to anaplasmosis (ELISA), blue tongue (BT, AGID)), bovine respiratory synctial virus (BRSV, VN)), bovine viral diarrhea (BVD, VN), brucellosis (ELISA), caprine arthritis and encephalitis (CAE, AGID), epizootic hemorrhagic disease (EHD, AGID), infectious bovine rhinotracheitis (IBR, VN), leptospirosis (MAT), and parainfluenza 3 (PI3, VN).

### Parasitology

Feces were collected using a lubricated, gloved finger inserted into the rectum, placed into plastic bags (Whirl-pac bags, Nasco, Inc., Fort Atkinson, WI), and refrigerated until analysis at the University of Idaho, Caine Veterinary Teaching Center, Caldwell, Idaho (CVTC) within 7 days after collection. Fecal material (1–5 g) was suspended in a saturated sucrose solution for 20 min following standard methods [19] and the eggs and larvae present were identified and quantified.

### Microbiology

Oropharyngeal swabs were collected using an oral speculum and guarded swabs (Fisherfinest Transport Swab, Fisher HealthCare, Houston, TX). After collection, the swabs were either immediately submitted to or held at 10 C and delivered within 48 hr to the CVTC. Swabs were plated on blood agar plates and incubated using standard bacteriological techniques [20]. Pasteurellacae were identified to biogroups [17, 21] and phenotypic characteristics were used to delineate the various *Mannheimia* species [22].

## Results

Management practices varied between pack and herd goats with pack goats reporting a higher degree of veterinary attention. Most pack goats were vaccinated against *Clostridium perfringens* C and D and *Clostridium tetani* (CDT) (9 of 11 herds, 82%) and dewormed with ivermectin (10 of 11 herds, 91%) annually. For herd goats, only 4 of 7 herds (57%) were vaccinated for CDT and 5 of 7 (71%) were dewormed with ivermectin, fenbendazole or a combination of ivermectin and fendendazole within the previous year. All goats sampled were judged to be in good physical and body condition with no obvious health problems.

Oropharyngeal swabs yielded isolates of one or more Pasteurellacae species from 43 of 48 (89.5%) pack goats and 41 of 43 (95.3%) for herd goats. The frequency of Pasteurellacae isolation was not significantly different between herd goats and pack goats (x^2^ = 2.5, df = 1, p = 0.41).

Within the two groups of goats, differences in the types and prevalence of specific Pasteurellacae species were found (Table 1). *Bibersteinia trehalosi* was found in 33 of 48 (68.8%) pack goats and 34 of 43 (79.1%) herd goats sampled with no significant differences between the herd types (x^2^ = 1.2, df = 1, p = 0.26). The majority of *B*. *trehalosi* isolates were biogroup 2 (21 of 33, 64% for pack goats and 27 of 34, 79% for herd goats). Very few *B*. *trehalosi* isolates were hemolytic (1 of 33, 3.0% and 7 of 34, 20.5% isolates from pack and herd goats, respectively).

**Table 1:** Occurrence of Pasteurellacae species from herd and pack goats from Idaho and Oregon, 2003.

Group type, No. and size (n)	*Bibersteinia trehalosi*		*Mannheimia haemolytica* [*M*. *glucosida*]		*M*. *ruminalis* [*M*. *varigena*]		*M*. species (untypeable)	
	#+/n	β hemolysis (#+/# isolates)	#+/n	β hemolysis(#+/# isolates)	#+/n	β hemolysis (#+/# isolates)	#+/n	β hemolysis (#+/# isolates)
Herd 1 (50)	11/12	7/11	2/12 [0/12]	2/2 [nd]	7/12 [1/12]	0/7 [1/1]	7/12	6/7
2 (9)	8/9	0/8	0/9 [0/9]	nd [nd]	1/9 [0/9]	0/1 [nd]	7/9	4/7
3 (7)	4/7	0/4	0/7 [0/7]	nd [nd]	0/7 [0/7]	nd [nd]	3/7	3/3
4 (4)	3/4	0/3	3/4 [3/4]	3/3 [2/3]	0/4 [0/4]	nd [nd]	2/4	1/2
5 (2)	2/2	0/2	1/2 [0/2]	1/1 [nd]	0/2 [0/2]	nd [nd]	1/2	1/1
6 (2)	0/2	Nd	1/2 [0/2]	1/1 [nd]	0/2 [0/2]	nd [nd]	0/2	nd
7 (7)	7/7	1/7	7/7 [1/7]	7/7 [1/1]	2/7 [0/7]	1/2 [nd]	1/7	0/1
Pack 1 (6)	3/6	0/2	0/6 [3/6]	nd [3/3]	0/6 [0/6]	nd [nd]	2/6	1/2
2 (2)	0/2	nd	0/2 [0/2]	nd [nd]	0/2 [0/2]	nd [nd]	0/2	nd
3 (4)	4/4	0/4	0/4 [0/4]	nd [nd]	0/4 [0/4]	nd [nd]	2/4	2/2
4 (6)	6/6	0/6	0/6 [5/6]	nd [5/5]	0/6 [0/6]	nd [nd]	0/6	nd
5 (5)	1/5	0/1	0/5 [0/5]	nd [nd]	1/5 [nd]	0/1 [nd]	0/5	nd
6 (7)	6/7	1/6	0/7 [0/7]	nd [nd]	0/7 [2/7]	nd [2/2]	4/7	4/4
7 (4)	3/4	0/3	0/4 [0/4]	nd [nd]	0/4 [0/4]	nd [nd]	1/4	1/1
8 (4)	2/4	0/2	0/4 [0/4]	nd [nd]	1/4 [0/4]	1/1 [nd]	2/4	2/2
9 (3)	3/3	0/3	0/3 [0/3]	nd [nd]	0/3 [0/3]	nd [nd]	1/3	1/1
10 (4)	4/4	0/4	0/4 [0/4]	nd [nd]	1/4 [0/4]	1/1 [nd]	1/4	0/1
11 (3)	2/3	0/2	0/3 [1/3]	nd [1/1]	0/3 [0/3]	nd [nd]	1/3	0/1

nnd, not determined.

*Mannheimia haemolytica* was not found in any of 48 pack goats, but was isolated from 14 of 43 herd goats (33%) (Table 1). Overall frequency of *Mannheimia* spp. isolation was significantly different between groups (x^2^ = 9.8, df = 1, p = 0.002). Isolates from both groups of goats were predominantly the unnamed *M*. species. Low numbers of *M*. *glucosida*, *M*. *ruminalis*, and *M*. *varigena* were found in both groups of goats. Most of the isolates of untypeable *Mannheimia* spp. [22] from pack and herd goats were hemolytic (22 of 26, 84.6% and 36 of 54, 66.7%, respectively). Based on biogrouping, most isolates from pack goats (20 of 26 isolates, 77%) and herd goats (40 of 54 isolates, 74%) were of high to moderate disease potential for bighorn sheep [21].

The majority of animals and herds had low to no titers to most pathogens assessed. No goats had antibodies against Anaplasmosis, IBR, or Leptospirosis. One herd goat was seropositive for *Brucella ovis* and three herd goats were seropositive to BVD and PI3. Antibodies to BT and EHD were prevalent in pack goats (25 of 48, 52.1%, seropositive to BT and 26 of 48, 54.2%, seropositive to EHD) but not in herd goats (2 of 41, 4.8% seropositive to both viruses) (x^2^ = 23.2, df = 1, p = 0.001) (Table 2). Animals with positive titers to BT and EHD came from 9 of 11 (82%) pack goat herds and 1 of 7 (14%) herd goat herds. Antibodies to CAE were found in 7 pack goats in 5 herds and 7 herd goats in 3 herds with the majority of seropositive animals from 2 pack goat herds and 1 herd goat herd.

**Table 2:** Occurrence of antibodies to selected viruses and fecal flotation for parasites in herd and pack goats from Idaho and Oregon, 2003.

Group type	Herd # and size (n)	Bluetongue(#+/n)	CAE(#+/n)	EHD(#+/n)	Coccidia(#+/n)				Strongyles(mean eggs/gram)
					High	Moderate	Low	0-Few	
Herd (H)	1 (50)	0/12	5/12	0/12	7/10	3/10			13.2
	2 (9)	0/9	1/9	0/9		2/9	3/9	3/9	206.8
	3 (7)	0/7	0/7	0/7		4/7	2/7	1/7	95.4
	4 (4)	0/4	0/4	0/4				4/4	168.8
	5 (2)	0/2	0/2	0/2	2/2				1.0
	6 (2)	nd	nd	nd				2/2	0.5
	7 (7)	2/7	1/7	2/7		1/7	2/7	4/7	3.4
Total		2/41	7/41	2/41	9/41	10/41	7/41	14/41	82.1
Pack (P)	1 (6)	2/6	1/6	2/6		3/6	2/6	1/6	408.3
	2 (2)	0/2	0/2	1/2			1/2	1/2	162.5
	3 (4)	3/4	1/4	4/4			1/4	3/4	58.0
	4 (6)	3/6	0/6	3/6			2/6	4/6	1.3
	5 (5)	2/5	0/5	2/5				5/5	0.0
	6 (7)	6/7	3/7	5/7			2/7	5/7	33.6
	7 (4)	2/4	0/4	2/4			1/4	3/4	0.0
	8 (4)	2/4	4/4	2/4		1/4	2/4	1/4	120.5
	9 (3)	3/3	1/3	3/3				3/3	28.0
	10 (4)	2/4	0/4	2/4				3/3	63.7
	11(3)	0/3	0/3	0/3		1/3	1/3	1/3	2.7
Total		25/48	7/48	26/48	0/47	4/47	12/47	30/47	85.4

**Fig 1:**
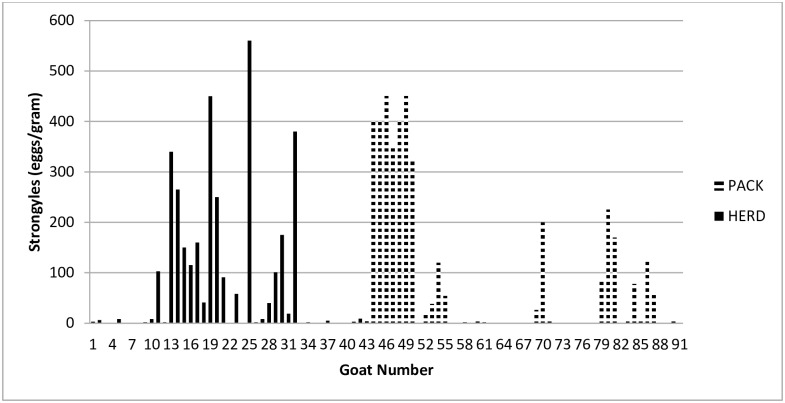
Histogram of fecal flotation results for *Strongyle* spp. in herd and pack goats.

Fecal samples were collected from 47 pack goats and 41 herd goats (Table 2). Only herd goats had individual animals with high or moderate levels of coccidia oocysts (19/40), while pack goats tended to have low to few coccidia oocysts (42/46). Goats in 9 herds (6 pack and 3 herd) averaged greater than 30 eggs/gram for *Strongylus* spp. and 17/47 (36%) pack and 16/41 (39%) herd goats had greater than 30 eggs/gram (Fig 1). Seven pack goats and 2 herd goats had low numbers of *Nematodirus* spp. ova. Eggs of *Trichuris* spp. were found in 4 pack goats and 5 herd goats with only 1 pack goat having a high egg count.

## Discussion

The goats in this study were in good health based on physical appearance and examination. Most goats sampled spent the majority of time in pastures; however goats were classified based on the owner’s use of the animals. Although the number of goat herds sampled was limited and herd size was generally small, all goats present on a premise were sampled with one exception. Therefore, the results were considered representative of the goats on each premise allowing comparisons between goat herds and herd type.

Management practices in these goat herds are likely typical of those in other areas. In general, the pack goats received more veterinary attention than herd goats, possibly reflecting the smaller numbers of goats owned and the degree of attachment between the owner and the goats. Despite herd type and management practices, most goats harbored some parasites and had exposure to various pathogens, all of which have been reported previously in domestic goats [3, 4, 17].

Parasites were present, but were considered to be of low consequence with the exception of some individuals from a few herds. Strongyles were the most common parasite found, with some herds having several goats shedding large numbers of ova. The presence of strongyle type and *Trichuris* spp. ova was not expected given the deworming programs in most herds and may reflect heavy pasture contamination with ova or inappropriate deworming dose, frequency or type. None of the gastro-intestinal parasites found in these goats were considered to be a severe consequence for BHS and all have been documented in BHS previously. Goat lungworms (*Muellerius* spp.) were not examined in this study and may constitute an additional risk to BHS [16].

The taxonomy of bacteria in the family Pasteurellaceae, first described in 1981, has undergone numerous changes, especially, but not limited to members of *Pasteurella haemolytica* [23]. Early classifications used biotypes “A, 3, and T” based upon serology and the ability to utilize trehalose sugar as a fermentation substrate. However, the “T” group is now known as *Bibersteinia trehalosi* [24], and serology is no longer recognized as a reliable method for identification [25]. Extensive changes were made to the taxon *Pasteurella haemolytica* when a phenotypic biovariant typing system was devised to accommodate wildlife isolates that varied from many domestic animal strains [21]. Using more modern genetic technology and redefining the genus *Pasteurella*, a new genus, *Mannheimia*, with five named species and two unnamed groups, was identified [26] using phenotypic characteristics [22] as *Mannheimia* spp. These techniques were used to classify the *M*. *haemolytica* isolates into *M*. *glucosida*, *M*. *verigena* and *M*. *ruminalis* (Table 1).

Herd goats had a higher prevalence of *Mannheimia* spp. isolations than pack goats. In general, hemolytic biovariants and isolates with higher disease potential [21] were found in herd goats compared to pack goats. In addition, most isolates were hemolytic which may correlate with virulence [27] in Pasteurellacae from a variety of host species [28]. Hemolysis was also highly associated with *ltkA* in over 50 isolates of *Bibersteinia* spp. in BHS [29].

Based on serology, pack goats had higher prevalence of antibodies to BT, EHD, and CAE than herd goats, but the time and place of exposure is unknown. No evidence of clinical disease associated with any of these agents was seen in the sampled animals. The presence of CAE was not unexpected, but the prevalence was higher than anticipated. CAE can be controlled in goats by whole herd testing and management, but it is time consuming. For pack goats that are expected to perform under load, it may be important to establish CAE free herds or ensure that individuals used for packing are CAE free. The generally older age pack goats may have had more exposure opportunities than the generally younger age herd goats.

Both pack and herd goats in this study were found to have respiratory bacteria which have been associated with pneumonia in BHS [17, 21]. The prevalence of Pasteurellacae isolated from domestic goats in this study is comparable to other reports [17, 30] and these results might help to define the typical oropharyngeal bacterial flora of domestic goats. Differences between pack and herd goats in this study are likely due to differences in age structure, herd size and the extent of interaction between goats from different sources.

Of the bacteria isolated from these goats, several biogroups are of concern for wildlife biologists that manage free-ranging BHS populations. Numerous published reports and circumstantial evidence suggest that contact with domestic sheep may lead to pneumonia in BHS [31, 32, 33, 34, 35, 36]. Under experimental conditions contact between domestic goats and BHS does not appear to be as problematic as contact with domestic sheep [14] but co-pasturing of domestic goats and BHS has resulted in pneumonia and death in BHS [16]. Contact between BHS and domestic goats under field conditions has been documented [10, 36, 37]. Infectious keratoconjuntivitis (pink eye) has been transmitted from domestic goats to BHS under range conditions [37] and *Mannheimia* spp. have been shown to be shared between BHS and feral goats, although the sharing was limited between three animals and did not appear to be involved in the large BHS die-off occurring in the area [10].

At the time this study was completed, the importance of *Mycoplasma* spp. for BHS was not recognized and minimal diagnostic options were available for this group of organisms. Since then, *M*. *ovipneumonia* has been found to be associated with population limiting disease in bighorns [13] and is currently recognized as a major factor in lamb pneumonia. In addition, *M*. *ovipneumonia* has been recognized in domestic sheep with respiratory disease [38] and domestic goats [39].

Recognition of the potential risk of contact between BHS and domestic goats and the potential for pathogen exchange is critical to minimize the risk of disease transmission between the two species [40]. For goats that are used for weed control or as pack animals in BHS habitat, appropriate management practices should be used to minimize the risk of interactions between goats and BHS [40]. Recommendations for management of pack goats should include avoiding direct contact between goats and BHS, the use a tether or lead rope at all times when in the presence of free-ranging BHS, and keeping goats under close control when in areas in which BHS could be present. Parasite control is highly recommended as a best management practice and should be required prior to use of goats in BHS habitat to minimize the risk of parasite transmission to BHS.

For herd goats that are used for weed management in BHS habitat, management recommendations should include avoiding direct contact between goats and BHS, deworming on a regular basis, and minimizing the introduction of new animals into the herd. The use of herders and dogs on site to manage the movements of goats and to haze BHS that come in close proximity to domestic goats is highly recommended.

If grazing by goats is effective in the elimination of noxious weeds, the use of goats for weed control in BHS habitat may be helpful in restoring range conditions but should include temporal and spatial separation between goats and BHS [40]. If goats are not or are only marginally effective for elimination of noxious weeds in BHS habitat, the potential risk of disease transmission from domestic goats to BHS is too high to consider the widespread use of domestic goats for this purpose.

Domestic goats are increasing in numbers in the United States and are part of small hobby farms, niche markets for meat and milk, and packing [1, 2, 41]. With the increase in the numbers and the use of goats, it is prudent for goat owners and wildlife managers to consider the potential impact of contact between domestic goats and BHS. It is hoped that the information from this study will allow management decisions that will minimize the risk of potential pathogens between domestic goats and BHS and allow the use of domestic goats within acceptable temporal and spatial separation parameters.
